# Foundation Model Artificial Intelligence for Electrocardiogram Detection of Rheumatic Mitral Stenosis

**DOI:** 10.64898/2026.10.12.26365300

**Published:** 2026-10-14

**Authors:** Raghav Sriram, Ivan Nenadic, Zak Loring, Marat Fudim, Anthony J. Mazella, Venkatesh L. Murthy, Anil K. Gehi

## Abstract

**Background:**

Rheumatic mitral stenosis (RMS) disproportionately affects low-resource settings where echocardiography is limited. We evaluated foundation model (FM) feasibility for RMS detection using limited, indirectly labeled ECGs.

**Methods:**

An ECG-FM was fine-tuned using 804 RMS-positive ECGs per fold and externally validated across clinical and wearable lead configurations.

**Results:**

Internal AUROCs were 0.89, 0.87, and 0.87, for 12-Lead, 6-Lead, and 1-Lead respectively. External AUROCs were 0.82, 0.80, and 0.77. Performance remained comparable across demographic subgroups.

**Conclusions:**

FM fine-tuning enables RMS detection using limited ICD-derived labels, supporting further evaluation of reduced-lead ECG screening and echocardiography triage in resource-constrained settings.

